# Bibliography

**Published:** 1887-03

**Authors:** 


### BIBLIOGRAPHY.

CHEMICAL LECTURE NOTES; taken from Prof. C. O. Curtman's lectures at St. Louis College of Pharmacy; By H. M. Whelpley, Ph. G., Quiz-Master of Chemistry and Pharmacology in St. Louis College of Pharmacy. St. Louis, 1886, published by the author, 136 pages, cloth; $1.

This little work consists of a series of carefully prepared notes by Prof. Curtman, from which he delivered his course of lectures in the above named college. In '84 they were re-written and a variety of information was added, gathered from journals and other sources not readily accessible to the student, and thus they form a complete sylabus of the subjects taught. The author, Dr. Whelpley, obtained permission to copy them for use in his quiz class, and for publication; and having added various paragraphs himself, obtained from other sources, and having enlarged on subjects or heads but lightly touched on by the professor, he presents them in this shape for the use of students of pharmacy and of medicine. They will doubtless be of use to others, however, and to any one who wishes to refresh his memory on the subject. Price $1. Address author, 404 Market St., St. Louis.

---

A Reference Handbook of the Medical Sciences, embracing the entire range of scientific and practical medicine and allied science. By various writers. Illustrated by chromo-lithographs and fine wood engravings. Edited by Albert H. Buck, M. D., New York City. Volume IV. Bound in cloth, sheep, and half morocco, prices, $6.00, $7.00, and $8.00 per vol. New York: William Wood & Co.

We tender our acknowledgments to the publishers for the fourth volume of this valuable work. It has received so much commendation at the hands of the press that we feel that anything we could add would be mere superfluity. To say the work is a library within itself would be tautology. It is destined, we believe, to outlive the present generation, and to become a standard book of reference for many years.

